# Improving the HER Activity and Stability of Pt Nanoparticles by Titanium Oxynitride Support

**DOI:** 10.1021/acscatal.2c03214

**Published:** 2022-10-12

**Authors:** Milutin Smiljanić, Stefan Panić, Marjan Bele, Francisco Ruiz-Zepeda, Luka Pavko, Lea Gašparič, Anton Kokalj, Miran Gaberšček, Nejc Hodnik

**Affiliations:** † Department of Materials Chemistry, National Institute of Chemistry, Hajdrihova 19, 1000Ljubljana, Slovenia; ‡ Laboratory for Atomic Physics, Institute for Nuclear Sciences Vinča, University of Belgrade, Mike Alasa 12-14, 11001Belgrade, Serbia; § Department of Physical and Organic Chemistry, Jožef Stefan Institute, Jamova cesta 39, 1000Ljubljana, Slovenia; ∥ Jožef Stefan International Postgraduate School, Jamova cesta 39, 1000Ljubljana, Slovenia; ⊥ Faculty of Chemistry and Chemical Technology, University of Ljubljana, Večna pot 113, 1000Ljubljana, Slovenia; # University of Nova Gorica, Vipavska 13, 5000Nova Gorica, Slovenia; 7 Centre of Excellence for Low-Carbon Technologies, Hajdrihova 19, 1000Ljubljana, Slovenia

**Keywords:** hydrogen evolution reaction, platinum nanoparticles, titanium oxynitride, strong metal−support interaction, XPS, DFT

## Abstract

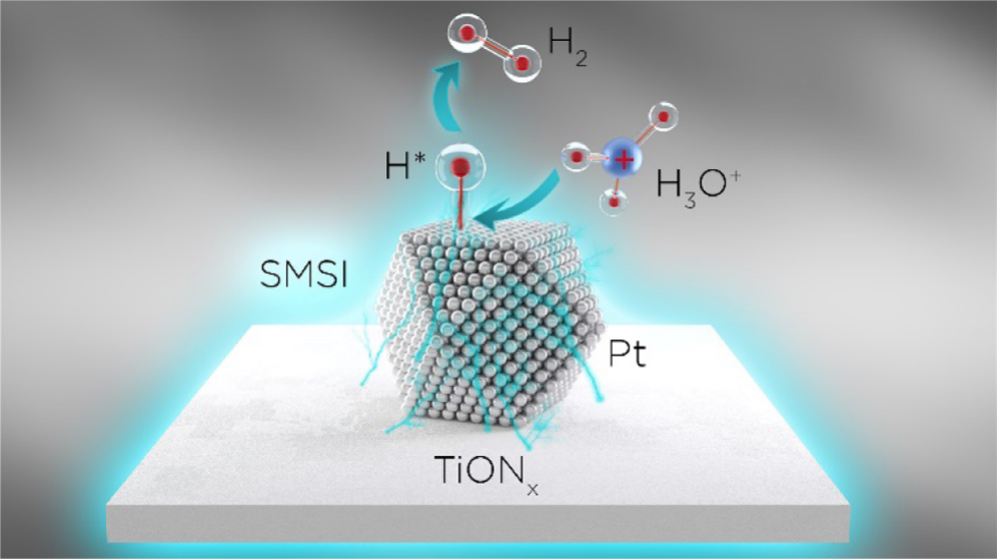

Water electrolysis powered by renewables is regarded as the feasible route for the production of hydrogen, obtained at the cathode side through electrochemical hydrogen evolution reaction (HER). Herein, we present a rational strategy to improve the overall HER catalytic performance of Pt, which is known as the best monometallic catalyst for this reaction, by supporting it on a conductive titanium oxynitride (TiON*_x_*) dispersed over reduced graphene oxide nanoribbons. Characterization of the Pt/TiON*_x_* composite revealed the presence of small Pt particles with diameters between 2 and 3 nm, which are well dispersed over the TiON*_x_* support. The Pt/TiON*_x_* nanocomposite exhibited improved HER activity and stability with respect to the Pt/C benchmark in an acid electrolyte, which was ascribed to the strong metal–support interaction (SMSI) triggered between the TiON*_x_* support and grafted Pt nanoparticles. SMSI between TiON*_x_* and Pt was evidenced by X-ray photoelectron spectroscopy (XPS) through a shift of the binding energies of the characteristic Pt 4f photoelectron lines with respect to Pt/C. Density functional theory (DFT) calculations confirmed the strong interaction between Pt nanoparticles and the TiON*_x_* support. This strong interaction improves the stability of Pt nanoparticles and weakens the binding of chemisorbed H atoms thereon. Both of these effects may result in enhanced HER activity.

## 1 Introduction

A transition to renewable and green energy and securing ever-increasing energy demands are among the greatest challenges imposed on modern society. Global energy systems based on fossil fuels cause significant environmental pollution, such as the exponential growth of anthropogenic CO_2_ emissions and consequent climate changes. Hydrogen economy^1,2^ was proposed half a century ago as a vision to circumvent these issues using hydrogen as the primary energy carrier instead of fossils. Hydrogen has the highest mass-energy density of any fuel, making it a potentially highly useful energy vector.^3^ In this scenario, the conversion of chemical energy of hydrogen into electricity is provided by fuel cells, zero-emission devices expected to find broad applications in both stationary and mobile power units. Currently, hydrogen production is one of the major obstacles to realizing the hydrogen economy, as steam reforming of methane derived from natural gas is still the dominant method of industrial-scale production, contributing significantly to the undesired CO_2_ footprint.^2^ Water electrolysis (WE) powered by renewables, such as wind and sun energy, has been recognized as a sustainable way to produce high-purity hydrogen.^4^ In WE, hydrogen is obtained at the cathode side through the electrochemical hydrogen evolution reaction (HER), while oxygen evolution reaction (OER) occurs at the anode. Due to its paramount importance for both fundamental and applicable aspects of electrocatalysis and physical chemistry in general, HER is one of the most intensely studied electrochemical processes.^5,6^ In acid solutions, the overall reaction can be written as
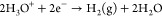
1Regarding the reaction mechanism, HER commences with the adsorption of a hydrogen atom in the so-called Volmer step
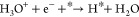
2where * stands for a free adsorption site and H* for the chemisorbed hydrogen atom. The reaction can proceed through the so-called Tafel step, in which chemical recombination of two adsorbed hydrogen atoms occurs
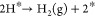
3Another option for reaction proceeding is electrochemical recombination or the so-called Heyrovsky step
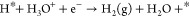
4According to Sabatier’s principle,^7^ the adsorption energy of H* can be considered as one of the main descriptors for the HER reactivity. Both traditional^8−10^ and revisited^11,12^ HER Volcano plots, which correlate experimentally measured exchange current density and calculated hydrogen adsorption energy, agree that Pt is the best monometallic catalyst for HER thanks to the close-to-optimal interaction with adsorbed hydrogen atoms. Due to its scarcity and high price, significant research efforts are engaged in designing alternative Pt-free electrocatalysts for HER (and other reactions where Pt is a catalyst). Promising results were reported with different materials based on abundant non-platinum-group metals, including, for example, sulfides,^13^ nitrides,^14^ carbides,^15^ and phosphides.^16^ However, their applicability is still challenged by rather inferior activity with respect to Pt and rapid deactivation, especially in corrosive acidic media.^17,18^ Therefore, rational usage of Pt and the design of advanced catalytic systems seems a viable pathway toward sustainable hydrogen production from water electrolysis. In addition, it is fundamentally exciting to see if the intrinsic HER activity of Pt can be enhanced, despite it being closest to the top of the HER Volcano plots.

Pt utilization can be increased by increasing the surface-to-volume ratio, which is why Pt electrocatalysts are composed of nanoparticles (NPs) dispersed on high-surface-area carbon supports.^19,20^ Carbon supports are the material of choice in electrocatalysis as they provide good electrical conductivity, a large surface area, and high chemical and electrochemical stability.^20^ Their downside is weak or almost no interaction with grafted nanoparticles, which can negatively impact the composite’s overall catalytic performance. Carbon supports do not provide any worthwhile effect on the intrinsic activity of metallic active centers, which can be achieved by modifying the electronic structure of active Pt sites.^20,21^ Moreover, the weak interaction between carbon and Pt nanoparticles allows the latter to migrate over the carbon surface during HER, making the composite prone to degradation via particle detachment, agglomeration, or coalescence. Both activity and stability of the electrocatalytic composites can be tuned by employing the so-called “strong metal–support interaction” (SMSI).^22−25^ SMSI can tune the electronic structure of the metallic active sites and affect their activity; at the same time, SMSI can trigger stronger binding or anchoring of active nanoparticles with support and thus improve stability. In that sense, different materials have been reported to provide merits of SMSI with supported Pt, including prefunctionalized carbons,^20^ carbides,^26^ and metal oxides.^25,27^

Our group has recently developed nitrogen-doped Ti oxide, also referred to as titanium oxynitride (TiON*_x_*), and applied it as the advanced support for Ir nanoparticles to form superior catalysts for OER. In their pioneering paper on TiON*_x_*,^28^ Bele and colleagues provided answers to a fundamental question about the applicability of this material as catalyst support by showing that it has similar electrical conductivity to carbon supports and was able to host well-dispersed small-sized Ir nanoparticles. More importantly, the first results on OER catalysis seemed quite promising as Ir/TiON*_x_* composite outperformed the benchmark catalysts (Ir black and IrO_2_) in terms of both activity and stability.^28^ In the following reports, Ir/TiON*_x_* catalysts were further improved by different approaches in synthesizing the composite, for example, by preparing nanotubular Ir/TiON*_x_*^29,30^ or by adding the high-surface-area carbon^31^ or reduced graphene oxide nanoribons^32^ to ensure better dispersion and higher surface area of TiON*_x_*. Such enhanced catalytic behavior was ascribed to the SMSI effect between TiON*_x_* and Ir nanoparticles.^30^ Density functional theory (DFT) calculations confirmed SMSI and showed that adhesion of Ir nanoparticles on TiON*_x_* was remarkably improved by the presence of surface N ions, which reduced the tendency of Ir nanoparticles to migrate and subsequently agglomerate.^30^ Such support effect is directly responsible for enhanced stability of Ir/TiON*_x_* during harsh OER conditions. In light of the mentioned benefits of using TiON*_x_* in OER catalysis, similar effects of this support could be expected in combination with other metallic nanocatalysts for different energy conversion reactions.

In this study, we investigate HER on a carbon-ceramic nanocomposite catalyst composed of Pt nanoparticles supported on TiON*_x_* embedded on reduced graphene oxide nanoribbons, further on denoted as Pt/TiON*_x_*. The structure of Pt/TiON*_x_* is characterized by X-ray diffraction (XRD) and transmission electron microscopy (TEM), while the electrocatalytic performance of the composite for HER is investigated in acidic media. To scrutinize the effect of the novel TiON*_x_* substrate, we performed analogous characterization and HER investigations with the Pt/C benchmark. X-ray photoelectron spectroscopy (XPS) was performed to study the effect of the TiON*_x_* substrate on the electronic states of supported Pt. DFT calculations were employed to verify the SMSI effect between the TiON*_x_* support and Pt nanoparticles and its impact on the binding of chemisorbed hydrogen atoms.

## 2 Experimental Section

### 2.1 Synthesis and Characterization of Pt/TiON*_x_* Composite

In the first step of synthesizing the Pt/TiON*_x_* composite, graphene oxide nanoribbons (GONR) were prepared.^33^ Briefly, 8 g of C-grade MWCNT (NTL) was added into a mixture of 1000 mL of sulfuric acid (Carlo Erba, 96%) and 110 mL of phosphoric acid (Merck, 85%) and stirred. Over the next 4 days, 8 wt equivalents of KMnO_4_ (8 × 8 g) was added to the mixture under stirring. The mixture was then quenched with ice, followed by adding 30% H_2_O_2_ until the color was changed from purple to yellowish. The supernatant was discarded, a portion of ultrapure water (resistivity 18.2 MΩ cm, obtained from Milli-Q Direct Water Purification System, MilliPore) was added, and the mixture was centrifuged for 30 min at 10 500 rpm (Sorvall LYNX 4000, Thermo Scientific). The obtained solid was redispersed in 5% HCl for 2 h to eliminate any residual metals. Afterward, the mixture was centrifuged for 30 min at 10500 rpm, followed by the supernatant decantation. The last cleaning step involved redispersing the GONR in ultrapure water and soaking it until the next day, followed by centrifugation at 10 500 rpm for 1 h to discard the supernatant. A total of five washing cycles in ultrapure water were conducted. Afterward, nanoribbons were redispersed in ultrapure water with a concentration of ∼20 g L^–1^ and treated in an ultrasonic bath (Iskra Sonis 4, Iskra) for 15 min to exfoliate the product. The suspension was then freeze-dried to obtain the dry product. In the next step, TiO_2_ coating on GONR was prepared. For this purpose, 0.1 g of dried GONR was mixed with 1 mL of propanol (Honeywell, 99.8%) solution containing 0.5 mmol of Ti isopropoxide (Aldrich, 97%). After mixing at room temperature, Ti isopropoxide was hydrolyzed by adding 0.2 mL of water (Milli-Q water, 18.2 MΩ cm). The obtained mixture was then dried in air at 50 °C. In the third step, a water solution containing 35 mg of Pt(NH_3_)_4_(NO3)_2_ (Alfa Aesar) (1 mL) was added to the dried mixture and lightly milled in a mortar at 50 °C until evaporation. Afterward, the mixture was thermally treated in a 90% NH_3_, 9.5% Ar, and 0.5% H_2_ mixture. The temperature was first increased at a rate of 2 °C min^–1^ to 250 °C for 2 h, then at a rate of 10 °C min^–1^ to 730 °C for 3 h, and then cooled to room temperature at a rate of 10 °C min^–1^. After the thermal treatment, the final Pt/TiON*_x_* material contained 18.4 wt % of Pt, according to the inductively coupled plasma-optical emission spectrometry (ICP-OES) analysis.^34^ The obtained Pt/TiON*_x_* composite was further characterized by XRD and TEM. XRD pattern was recorded using a D4 Endeavor, Bruker AXS diffractometer with Cu Kα radiation (λ = 1.5406 Å), and a Sol-X energy-dispersive detector. For the detailed microstructural investigation, a Cs probe corrected scanning transmission electron microscope (Jeol ARM 200 CF) with an attached Jeol Centurio EDXS system with 100 mm^2^ SDD detector and Gatan Quantum ER DualEELS system was used. For comparison, the same characterization was also performed for a commercial 20 wt % Pt/C catalyst purchased from Premetek (Figure S1 in the Supporting Information).

### 2.2 Electrochemical Characterization and HER Investigations

The Pt/TiON*_x_* powder was mixed with Milli-Q water at a concentration of 1 mg mL^–1^, and the mixture was exposed to an ice-cooled ultrasonic bath to obtain fine catalyst dispersion (∼20 min). The same procedure was used for the Pt/C benchmark sample. Working electrodes were prepared by drop-casting 25 μL of catalyst suspensions onto mirror-polished glassy carbon (GC) rotating disk electrodes (RDE) provided by Pine (geometric area of 0.196 cm^2^). After drying in a closed desiccator, the films were covered with 5 μL of Nafion (Sigma, 5% solution in a mixture of lower aliphatic alcohols and water) diluted in isopropanol (1/50) to ensure good adhesion. In the case of Pt/TiON*_x_* and Pt/C, 4.6 and 5 μg of Pt, as the active compound, were loaded on the working GC electrode.

Glass electrochemical cell and all accompanying components were cleaned daily by boiling in distilled water for at least 1 h, followed by extensive rinsing in Milli-Q water. Electrochemical measurements were performed in a conventional three-electrode arrangement. Ag/AgCl and a carbon rod were used as reference and counter electrodes, respectively. Since leakage of chlorides from Ag/AgCl can significantly impact the experiments, Ag/AgCl was isolated in the separate reference electrode compartment (REC) from the working electrode compartment (WEC) and connected *via* an electrolytic bridge. Such a setup effectively slows the diffusion of chloride impurities from REC to WEC;^35^ hence, the impact of chloride contamination in this work is eliminated. All potentials will be reported *versus* the reversible hydrogen electrode (RHE).

Electrochemical characterization and HER investigations were performed in argon-saturated 0.1 M HClO_4_ electrolyte (Merck, 70% HClO_4_) diluted with Milli-Q water. Prior to measuring activity, catalyst films were electrochemically activated by 200 fast voltammetric scans (300 mV s^–1^) in the potential range of 0.05–1.2 V_RHE_. This ensures reaching a stable response and full wetting of the catalyst layer. After activation, three cyclic voltammograms (CVs) in the potential range between 0.05 and 1.28 V_RHE_ (50 mV s^–1^) were recorded for the purpose of electrochemical characterization. CO stripping voltammetry was used to evaluate the electrochemical surface area (ESA) of Pt/C and Pt/TiON*_x_* catalysts. For that purpose, CO was introduced into the electrolyte for 1 min while the electrode potential was kept at 0.05 V_RHE_. Dissolved CO was removed from the electrolyte by purging with Ar for over 30 min to ensure that the voltammetric response originates only from adsorbed CO on the Pt surface (and not from dissolved CO). CO stripping voltammogram was then recorded in the potential window 0.05–1 V_RHE_ at a scan rate of 20 mV s^–1^, followed by another CV in the same conditions to confirm the successful removal of dissolved CO. HER activity was evaluated by recording polarization curves in the potential region between 0.2 and −0.1 V_RHE_ at a scan rate of 10 mV s^–1^. Electrochemical impedance spectroscopy (EIS) measurements were performed at HER overvoltage of 20 mV in the frequency range between 50 mHz and 100 kHz with an amplitude of 10 mV. For the stability test, catalysts were exposed to extensive cycling (5000 cycles, 100 mV s^–1^) in the same potential range used for HER activity evaluation.

### 2.3 X-ray Photoelectron Spectroscopy Measurements

X-ray photoelectron spectroscopy (XPS) measurements were performed with the Versa probe 3 AD (Phi, Chanhassen, MN) using a monochromatic Al Kα X-ray source at an operating voltage of 15 kV and an emission current of 3.3 mA. Powder samples were placed on double-sided Scotch tape. Spectra were acquired on a 1 × 1 mm^2^ analysis spot size. During the measurements, the charge neutralizer was on. High-resolution (HR) spectra were measured at a pass energy of 27 eV and the binding energy (BE) step of 0.1 eV. Every spectrum was acquired with at least 20 sweeps to improve the signal-to-noise ratio. The BE scale of XPS spectra was corrected using the C–C/C–H peak in C 1s spectra corresponding to adventitious carbon at BE of 284.8 eV. Data processing (including fitting) was performed with the MultiPak 9.0 software. The Shirley background subtraction was used for all measurements. Three main doublet peaks were identified in Pt 4f region for both Pt/C and Pt/TiON*_x_* samples corresponding to the Pt^0^, Pt(OH)_2_, and PtO. The spin–orbit splitting value was kept constant at 3.33 eV for all of the bands between Pt 4f_7/2_ and Pt 4f_5/2_ peaks. The full width at half-maximum (FWHM) was constrained between 1.0 and 1.1 eV for all bands, while the peak shape was chosen to be asymmetric.

### 2.4 DFT Calculations

DFT calculations were performed with the PWscf package from Quantum ESPRESSO^36,37^ using the generalized-gradient-approximation of Perdew–Burke–Ernzerhof (PBE)^38^ and D3 dispersion correction of Grimme.^39^ For calculations involving titanium oxynitride (TiON*_x_*) support, a GGA + *U* method^40,41^ was utilized. The *U* parameter for Ti ions (4.0 eV) was taken from our previous publication,^30^ where it was calculated self-consistently for TiON_*x*_ bulk using the hp.x code that utilizes the density functional-perturbation-theory scheme.^42^ Kohn–Sham orbitals were expanded in a plane-wave basis set with a kinetic energy cutoff of 50 Ry for the wave function and 575 Ry for the charge density. Core electrons were described with projector-augmented-wave (PAW) potentials^43^ taken from PSlibrary.^44^ Carbon support was modeled as a single graphene layer using calculated two-dimensional (2D) hexagonal unit cell parameters of *a* = *b* = 2.467 Å. For the TiON*_x_* support, we used a symmetric nonpolar Ti_1.5_ON(111) slab consisting of three Ti layers, terminated on both sides by an O/N layer that contains only 50% of O and N ions to maintain the Ti_1.5_ON stoichiometry.

The carbon support, here modeled as graphene, was simulated with a (7 × 7) supercell, and a (3 × 3) supercell was used for the TiON_*x*_ support. The lateral dimensions of these two hexagonal supercells are similar (*A* = *B* = 17.27 and 17.70 Å, respectively, where *A* and *B* are the lengths of the two supercells vectors). The supercells are large enough to accommodate modeled Pt NPs (see below). Brillouin zone integrations were performed with a gamma *k*-point and a Methfessel–Paxton smearing^45^ of 0.02 Ry.

Pt nanoparticles (NPs) were built to match experimentally observed shapes of truncated octahedrons as determined from the TEM analysis (Figure S2 in the Supporting information). We used two different Pt NPs composed of (111) and (100) facets, labeled as Pt⟨7,4⟩ and Pt⟨6,9⟩, where the *i* and *j* numbers in the Pt⟨*i*,*j*⟩ label indicate the number of surface atoms consisting the (111) and (100) facets, respectively. The reason for using two different nanoparticles is to increase the reliability of predictions (using an even higher number of different NPs would be even better in this respect). Pt NPs were adsorbed on TiON*_x_* in such a way as to continue the fcc stacking of the Ti(111) layers of TiON*_x_*. On graphene, different lateral positions of NPs were tested, and the most stable identified structure was further used. On these supported NPs, we modeled the chemisorption of hydrogen at different coverages ranging from a single H atom per NP to a fully covered NP. Pt⟨7,4⟩ and Pt⟨6,9⟩ consist of 38 and 55 atoms, respectively, out of which 32 and 42 are surface atoms. The average chemisorption energy was calculated as
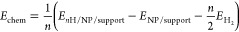
5where *E*_*n*H/NP/support_, *E*_NP/support_, and *E*_H_2__ are the total energies of a supported Pt NP system with *n* adsorbed H atoms, a bare supported Pt NP system, and isolated hydrogen molecule, respectively, calculated at 0 K without the zero-point energy (ZPE). Standard adsorption free energies were calculated as
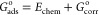
6where the *G*_corr_^o^ term includes the ZPE, thermal, and entropy contributions to the adsorption free energy at standard conditions. The free energy of the gas-phase H_2_ was calculated as
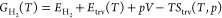
7where the “trv” subscript stands for translational + rotational + vibrational. *E*_trv_(*T*) thus represents the “trv” thermal energies at temperature *T* with ZPE included (this notation implies that *E*_trv_(0) = *E*_vib_(0) = ZPE, where *E*_vib_ designates vibrational energy). For gaseous H_2_, the roto-translational contributions were calculated using the rigid-rotor model and the ideal gas approximation (*pV = RT*). For catalysts and adsorbates thereon, only the vibrational contributions to thermal energy and entropy were considered, whereas the configurational entropy of adsorbates was neglected. The *pV* term was also neglected as it is negligible for solids. Hence
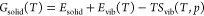
8where *E*_solid_ stands for either *E*_*n*H/NP/support_ or *E*_NP/support_ (cf. eq 5). To make vibrational calculations feasible, the *E*_vib_ and *S*_vib_ contributions to adsorption free energies were estimated from the simplified surrogate calculations of H adsorbed on Pt(111) and Pt(100) slabs. To this end, vibrational frequencies were calculated at the gamma q-point using the PHonon code^46^ from Quantum ESPRESSO. Vibrational frequencies below 100 cm^–1^ were raised to 100 cm^–1^ to correct for the breakdown of the harmonic oscillator model at low frequencies.^47^

The thermodynamic stability of different coverage of H atoms was determined with the adsorption surface free energy
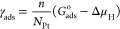
9where *N*_Pt_ is the number of surface Pt atoms of NP (γ_ads_ is hence normalized to a surface Pt atom) and Δμ_H_ is the H chemical potential measured with respect to the standard chemical potential of hydrogen (μ_H_2__^o^)
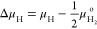
10which can be expressed as a function of electrode potential *U*
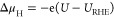
11where *U*_RHE_ is the reference electrode potential of the RHE electrode used in experimental measurements. With the above equations, we can predict the range of chemical potentials relevant to experimental conditions.

The adhesion of Pt nanoparticles on graphene and TiON_*x*_ supports was analyzed with adhesion energy (*E*_adh_) and electron charge density difference (Δρ(***r***))
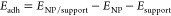
12and
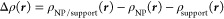
13where the subscripts NP/support, NP, and support stand for the supported NP, isolated NP, and bare support, respectively. For the calculation of *E*_adh_, the NP and support structures were relaxed, whereas for Δρ(***r***), the structures of NP and support were kept the same as in the NP/support system. In addition, a planar integrated electron charge density difference was also calculated by integrating Δρ(***r***) over the *xy* slices, i.e.
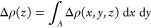
14where *A* is the area spanned by the 2D supercell.

## 3 Results and Discussion

### 3.1 Characterization of Pt/TiON*_x_* and Pt/C Catalysts

**Figure 1:**
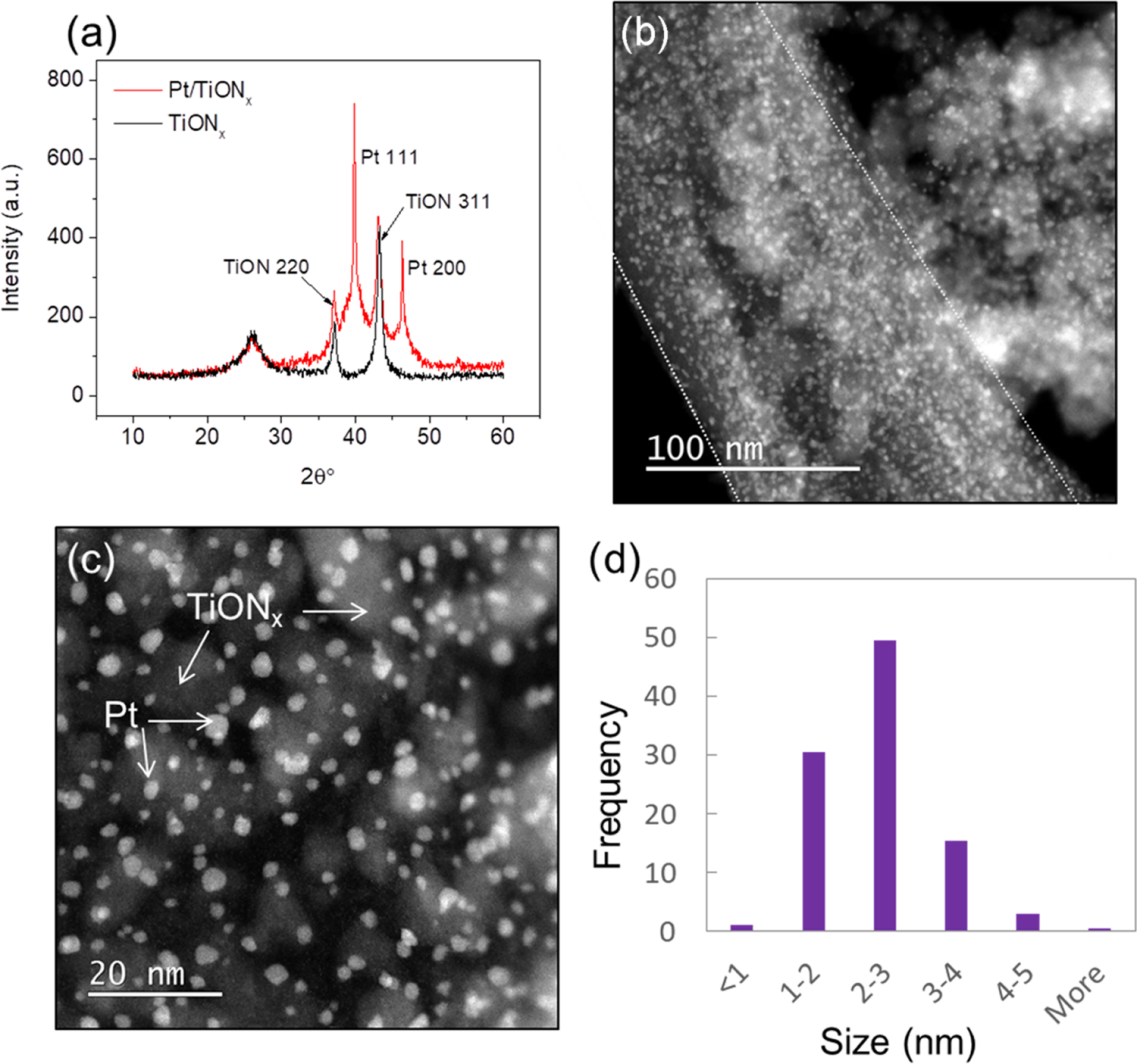
Characterization of a Pt/TiON*_x_* composite: (a) XRD spectra; (b) STEM imaging showing the overall structure of the sample; (c) high-magnification STEM imaging showing predominant anchoring of Pt NPs to TiON*_x_*; (d) particle size distribution.

Characterization of the novel Pt/TiON*_x_* composite was performed by XRD and TEM, and the obtained results are given in Figure 1. XRD pattern of the base TiON*_x_* support (black line, Figure 1a) contains diffraction peaks at 37.1 and 43.1° originating from the TiON*_x_* crystal structure (00-049-1325), while the reflections related to the initial TiO_2_ compound (01-073-8760) are absent, indicating the effective formation of TiON*_x_* during synthesis. In the case of Pt/TiON*_x_*, the same TiON*_x_*-related peaks are present in the XRD pattern (red line, Figure 1a), while the peaks appearing at 39.8 and 46.3° correspond to 111 and 200 reflections of Pt (JCPDS card 04-0802). Further characterization of Pt/TiON*_x_* was performed by TEM imaging (Figure 1b,c). Figure 1b shows a characteristic elongated structure of graphene nanoribbons (with a width of approximately 150 nm; emphasized by white dashed lines), covered with TiON*_x_* flakes (in a 20 nm size range) and decorated with small-sized and well-distributed Pt nanoparticles. The distribution of Pt nanoparticles between TiON*_x_* and carbon support is critical for SMSI, in the sense that Pt nanoparticles should be attached to TiON*_x_* (which can trigger SMSI),^30^ and not to carbon (which cannot induce SMSI). Close inspection of the higher-magnification scanning TEM (STEM) image (Figure 1c) reveals that Pt particles are indeed almost exclusively grafted on TiON*_x_*, which proves that the synthesis of the composite was conducted properly and that the mandatory prerequisite for SMSI is fulfilled. Analysis of particle size distribution (PSD) in Pt/TiON*_x_* (Figure 1d) shows that the Pt particles are generally below 5 nm in diameter, the majority being between 2 and 3 nm.

The same characterization was performed for the Pt/C benchmark sample (Figure S1). XRD spectra of Pt/C (Figure S1a) reveal the same Pt-related peaks as observed in Pt/TiON*_x_*, but the intensities and width of the peaks point out to slightly smaller particles. This is confirmed by TEM imaging (Figure S1b): the corresponding PSD shows a peak around 2 nm (Figure S1c). It should also be mentioned that the morphology of the Pt nanoparticles in the two samples is the same, i.e., in both spherical shapes with all lower Miller index facets can be found (Figure S2). Despite the slight difference in PSD, the two samples are still similar enough to enable a proper comparison of their HER activities.

Another important feature of the Pt/TiON*_x_* composite is the presence of Pt single atoms (SAs), which can be observed in STEM images taken at the highest magnifications. One representative example is given in Figure S3, where Pt SAs can clearly be distinguished as small bright dots. Single atoms are a hot topic in electrocatalysis, as they offer maximum metal utilization and intriguing catalytic performances. It is well known that SAs are intrinsically unstable; hence, their interaction with support is essential in preventing their aggregation.^48,49^ This means that the presence of SAs anchored on TiON*_x_* is already a sign of possible SMSI. However, as shown below, they do not affect the HER performance in the present case.

### 3.2 Electrochemical Characterization and HER Investigations

**Figure 2:**
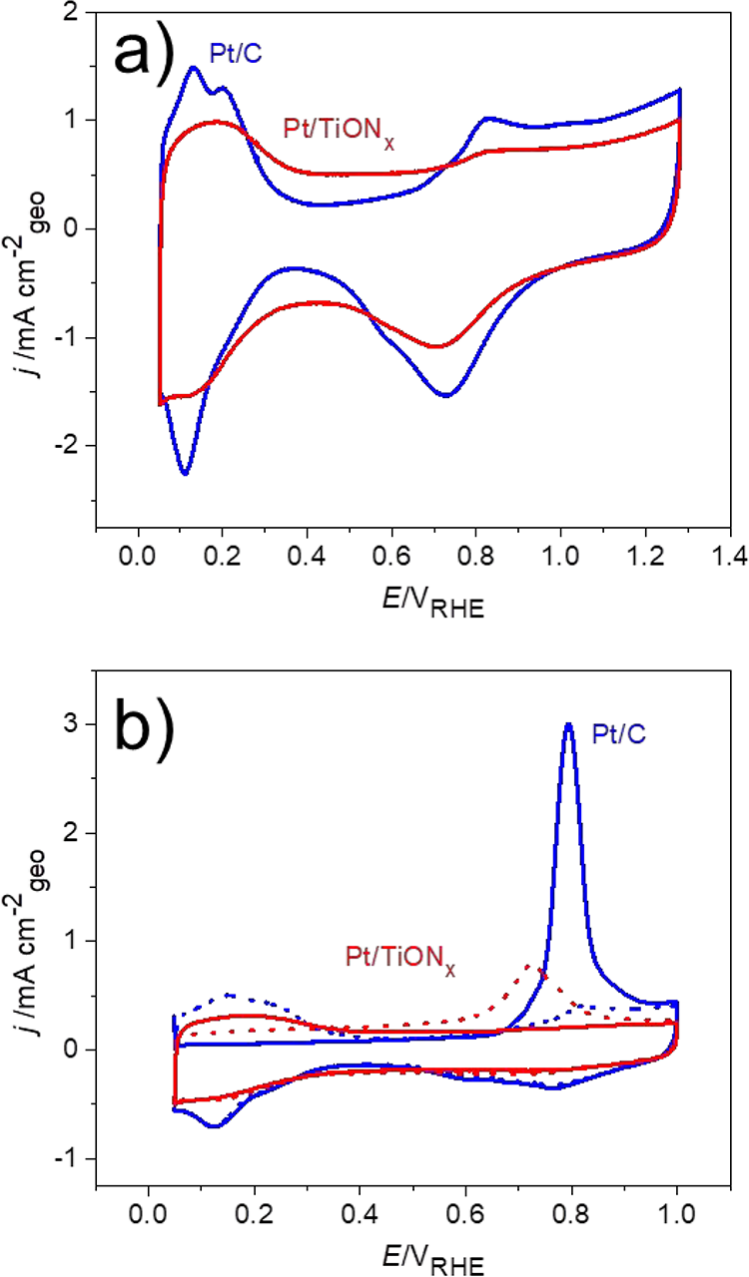
(a) Cyclic voltammograms of the Pt/C and Pt/TiON*_x_* catalysts (Ar-saturated 0.1 M HClO_4_, 50 mV s^–1^, Pt loadings were 5 μg for Pt/C and 4.6 μg for Pt/TiON*_x_*); (b) CO stripping voltammetry (full lines) and subsequent voltammograms (dotted lines) for Pt/C and Pt/TiON*_x_* (0.1 M HClO_4_, 20 mV s^–1^, Pt loadings were 5 μg for Pt/C and 3.7 μg for Pt/TiON*_x_*).

Electrochemical characterization of the Pt/TiON*_x_* and Pt/C samples was performed by cyclic voltammetry (CV) and CO stripping in 0.1 M HClO_4_ (Figure 2). As in the case of Pt/C, the CV of the Pt/TiON*_x_* composite shows all well-known electrochemical fingerprints of supported Pt nanoparticles, Figure 2a. These include the region of hydrogen underpotential adsorption (H_upd_) at potentials below 0.4 V_RHE_ and Pt redox processes at potentials above 0.5 V_RHE_.^50^ Overall, the CV of Pt/TiON*_x_* confirms the successful synthesis of the composite and electrochemical accessibility of the grafted Pt. The electrochemical surface area (ESA) of electrocatalytic materials is an important metric for properly comparing their intrinsic activities. For Pt-based catalysts, conventional methods for ESA evaluation are based on the charge corresponding to H_upd_ process or the oxidation of the adsorbed CO (CO stripping voltammetry).^50,51^ ESA values obtained by integrating H_upd_ peaks were 86 and 55 m^2^ g_Pt_^–1^ for Pt/C and Pt/TiON*_x_*, respectively. Similar ESA values of 89 and 61 m^2^ g_Pt_^–1^ were extracted from the CO stripping voltammetry for Pt/C and Pt/TiON*_x_*, respectively, Figure 2b. We note that the range of the measured ESA values is in line with the theoretically calculated ESA values of the particle with the sizes of 2–4 nm.^52^ Values obtained from the CO stripping will be used to evaluate the intrinsic HER activities of the two samples.

Interestingly, the CO stripping revealed that Pt/TiON*_x_* is more active (it oxidizes CO at lower potentials) than Pt/C for this reaction. It was shown earlier that particle size could play an important role in the reactivity of Pt/C for CO stripping,^53,54^ where larger particles with more surface defects are more active than the smaller ones. Such an effect was shown to be substantial for the Pt nanoparticles with diameters ranging between 1 and 30 nm.^54^ However, in our case, the difference in particle size between the two samples is not that substantial and thus unlikely to play a critical role in CO oxidation activity trends. More likely, this could be another indicator of SMSI that can contribute to CO oxidation activity in two ways: (i) through bifunctional effect, where the role of TiON*_x_* is to provide adsorbed OH^–^ ions, which are involved in CO oxidation,^54,55^ at lower potentials than on Pt nanoparticles; (ii) through tuning the adsorption energy of strongly bonded CO on Pt active sites.

**Figure 3:**
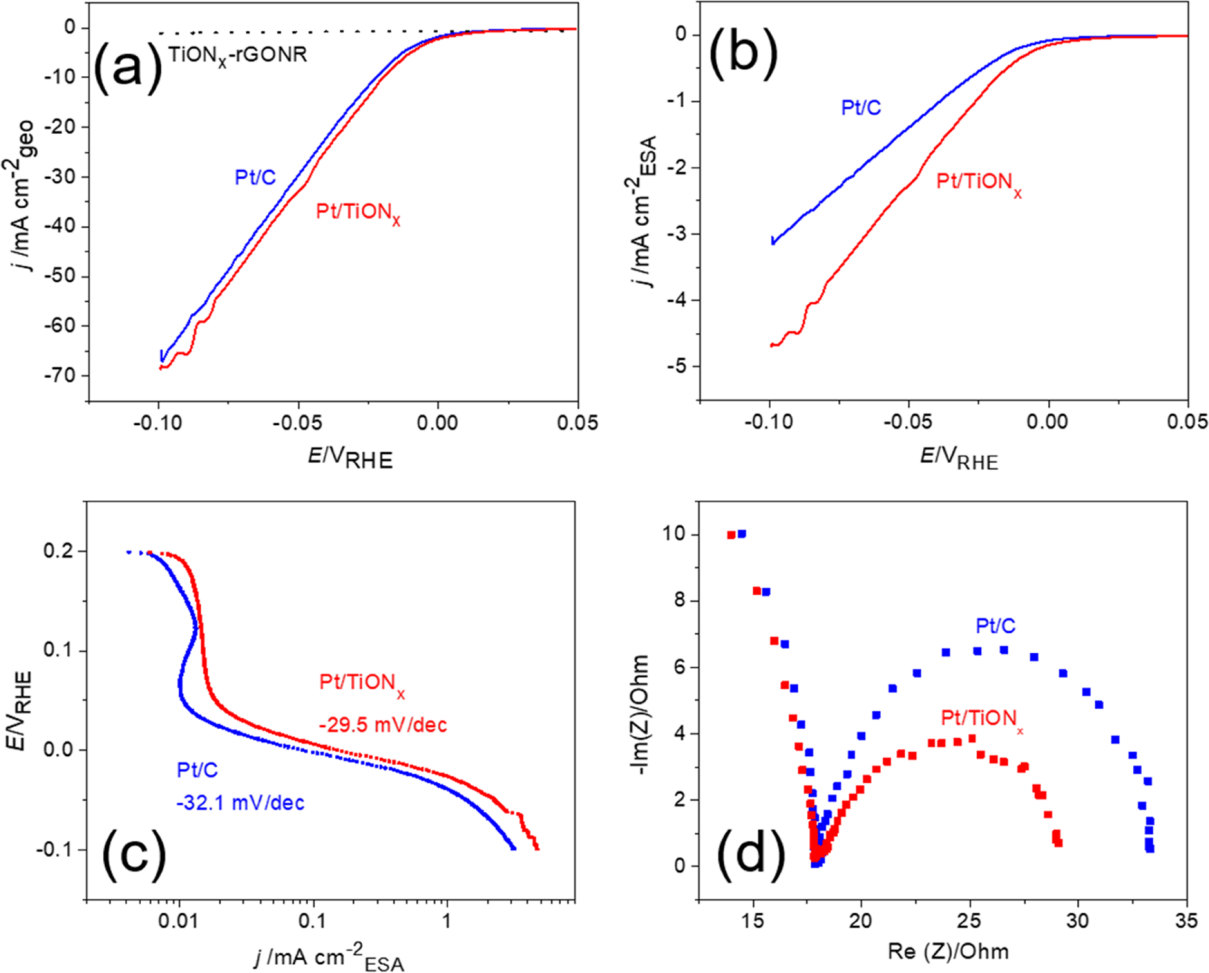
Comparison of the HER activity of the Pt/C and Pt/TiON*_x_* catalysts: (a) HER polarization curves (0.1 M HClO_4_, 10 mV s^–1^); (b) intrinsic HER activity given with respect to ESA; (c) Tafel slope analysis derived from polarization curves from (a); and (d) electrochemical impedance spectroscopy (−20 mV_RHE_, 50 mHz to 100 kHz, amplitude 10 mV).

Investigations of the catalytic performance of Pt/TiON*_x_* and Pt/C for HER in the acidic electrolyte are given in Figure 3. HER polarization curves for Pt/C and Pt/TiON*_x_* are presented in Figure 3a, along with the polarization curve for HER on TiON*_x_*-GONR substrate to show its inactivity for HER. More importantly, it can be seen that the novel Pt/TiON*_x_* composite is slightly more active than the benchmark Pt/C for HER in acid media, which is important when considering that Pt is the best HER catalyst. A comparison of the intrinsic activities of these two samples by taking the ESA into account is shown in Figure 3b. In this case, the difference in HER activity becomes more pronounced in favor of Pt/TiON*_x_*. To further compare intrinsic activities, turnover frequencies (TOF) were calculated^56,57^ based on the charge corresponding to H_upd_ and currents measured at HER overpotential of 50 mV. TOF values of 6.43 and 12.15 s^–1^ were obtained for Pt/C and Pt/TiON*_x_*, respectively, confirming the intrinsic improvement in HER activity of Pt when TiON*_x_* is used as a substrate. Mass activities of the two samples at the overvoltage of 50 mV are 1410 and 1160 A g_Pt_^–1^ for Pt/TiON*_x_* and Pt/C, respectively, which is an increase of nearly 20% (not shown). The corresponding Tafel plot (Figure 3c) exhibits a slightly lower slope of −29.5 mV dec^–1^ for Pt/TiON*_x_versus* −32.1 mV dec^–1^ fitted for Pt/C. Tafel slopes of about −30 mV dec^–1^ are otherwise common for Pt-based catalysts in acid media and correspond to the Volmer–Tafel pathway.^17,25,58^ Electrochemical impedance spectroscopy, Figure 3d, shows that charge transfer resistance is lower in the case of Pt/TiON*_x_* than for the Pt/C benchmark, agreeing with the improved HER activity of the novel composite catalyst.

**Figure 4:**
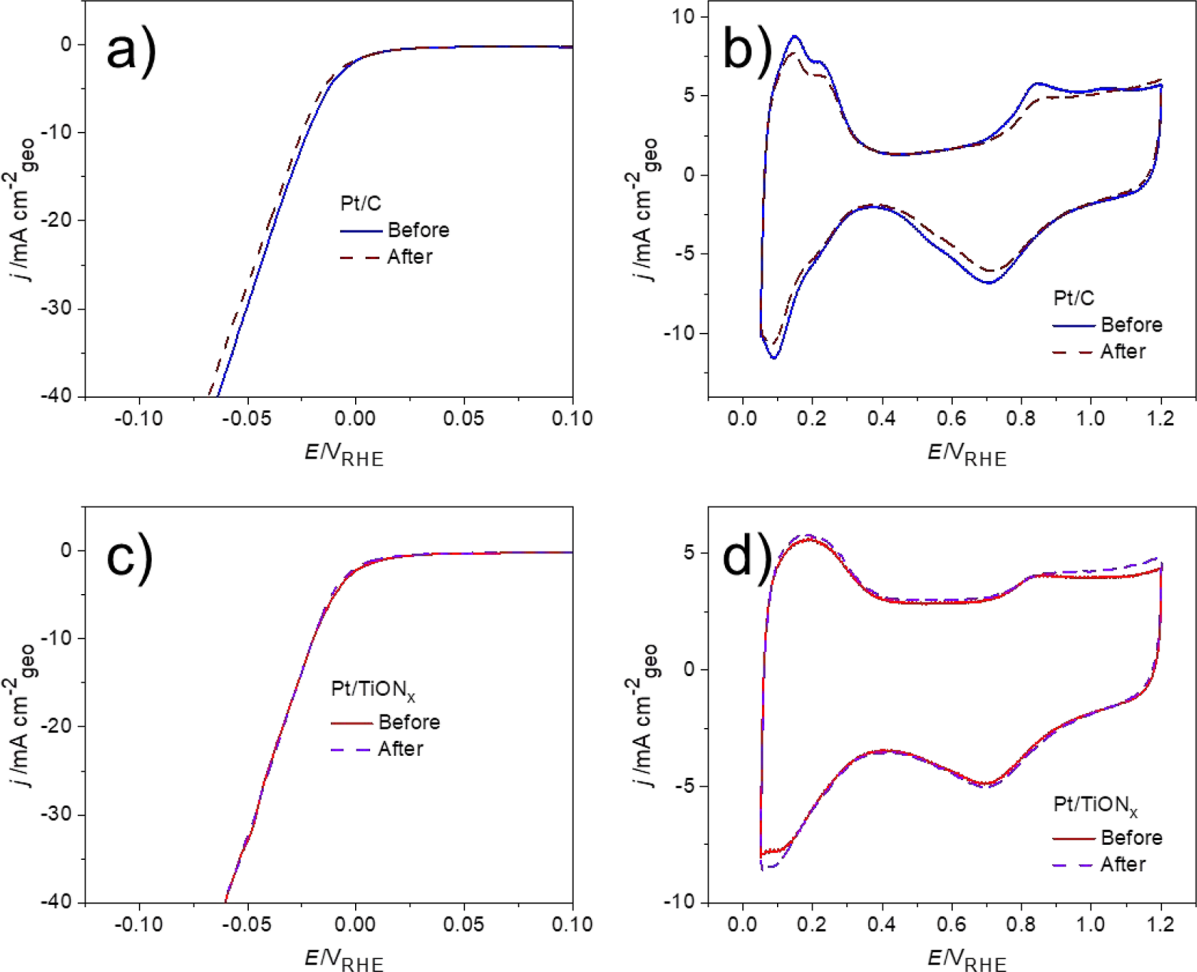
Stability test for the Pt/C and Pt/TiON*_x_* catalysts performed by cycling in the potential region between −0.1 and 0.2 V_RHE_ (5000 cycles, 100 mV s^–1^, 0.1 M HClO_4_): (a, c) HER polarization curves (10 mV s^–1^) before and after the stress test for Pt/C and Pt/TiON*_x_*, respectively, and (b, d) the corresponding cyclic voltammograms (200 mV s^–1^) of Pt/C and Pt/TiON*_x_*, respectively.

The stability of the electrocatalytic materials is another basic yet critical requirement for their applicability. Usually, the stability of the electrocatalysts is probed by chronopotentiometric/chronoamperometric tests and by exposing the catalysts to extensive cycling in the relevant potential window since different degradation mechanisms can occur under cycling and steady-state operation. Testing powdered catalysts in the form of thin films in the RDE setup using a constant potential or current during gas-evolving reactions (such as HER) is challenging due to the formation of microscopic bubbles which remain attached to the active sites.^59^ In that way, bubbles block the active sites from further exposure to the electrochemical environment, which may lead to false conclusions about their intrinsic performance. This matter was studied in detail in recent work on oxygen evolution on Ir catalysts.^60^ Exposing the Ir disk electrode to ultrasonication during OER provided direct proof that shielding of the active sites by evolved oxygen microbubbles is responsible for pronounced and rapid activity drop. Unfortunately, this effective methodology for bubble removal cannot be extended to thin-film powdered catalysts, as exposing them to ultrasonication will lead to the mechanical detachment of the catalyst layer from the glassy carbon substrate. Therefore, instead of using chrono-methods, the Pt/C and Pt/TiON*_x_* samples were subjected to extensive potential cycling under HER conditions. Since the formation of the microscopic bubbles cannot be fully eliminated, we took care to minimize its impact on the stability test as much as possible. We have set the lower potential limit to −0.1 V_RHE_ and the scan rate to 100 mV s^–1^ to avoid extensive bubble generation and to reduce the bubble accumulation time. At the same time, the upper potential limit was set at 0.2 V_RHE_ to provide more time for the effective removal of the trapped bubbles during the oxidation of previously evolved hydrogen. In general, the results of this test (Figure 4) reveal that both catalysts exhibit good stability; however, Pt/C is still less stable than Pt/TiON*_x_*. In the case of the Pt/C benchmark, the applied stress test caused a slight loss of the HER activity (Figure 4a) coupled with slight decay of Pt ESA (Figure 4b). In contrast, both HER activity (Figure 4c) and Pt ESA (Figure 4d) of Pt/TiON*_x_* remained fully stable.

Electrochemical stability and degradation of Pt/C catalysts is a widely studied topic due to their importance for fuel cells and electrolyzers. Different degradation mechanisms are well established depending on the catalyst treatment; these include Pt dissolution, followed by redeposition onto existing particles (Ostwald ripening), agglomeration, carbon corrosion, and particle detachment.^61^ At conditions used for the HER stress test (cycling between 0.2 and −0.1 V_RHE_), Pt dissolution, Ostwald ripening, and carbon corrosion can be excluded, leaving migration with subsequent agglomeration and particle detachment as remaining options. Indeed, these two mechanisms were proposed to occur during prolonged HER on Pt/C precisely due to the weak interaction between Pt nanoparticles and carbon support.^17,62^ Therefore, the improved HER stability of Pt/TiON*_x_* could be linked with SMSI and stronger binding of Pt nanoparticles to the TiON*_x_* support. Overall, it can be concluded that Pt/TiON*_x_* outperforms Pt/C in terms of both HER activity and stability.

Now we proceed with discussing the origin of the improved HER catalysis on Pt/TiON*_x_* with respect to Pt/C. To come down to the effect of the TiON*_x_* substrate and SMSI, we will first discuss a few possible nonintrinsic factors. As mentioned earlier, besides nanoparticles, the Pt/TiON*_x_* sample contains single atoms. Single atoms are a popular topic in catalysis as they offer maximum metal utilization, lack of metal–metal bonds, and the opportunity to tune their activity via the support effect.^48,63^ Pt SAs anchored on different supports have been explored in HER catalysis, and usually, improved activity was connected with SMSI.^17,64−66^ The coexistence of nanoparticles and SAs in the sample makes it difficult to unambiguously pinpoint which of the two is active. To investigate if Pt SAs present in the Pt/TiON*_x_* sample are the active sites for HER, we prepared a sample with Pt SAs on TiON*_x_* support (0.3 wt % Pt, further labeled as Pt-SA/TiON*_x_*). We took special care that Pt-SA/TiON*_x_* sample did not contain any Pt nanoparticles or even few-atom clusters, which are known to catalyze HER. The absence of nanoparticles and the presence of exclusively Pt single atoms in Pt-SA/TiON*_x_* were confirmed by TEM imaging (Figure S4a). The activity of Pt-SA/TiON*_x_* for HER was very low with respect to Pt/TiON*_x_* (Figure S4b) and similar to the activity of bare TiON*_x_* support. When normalized to the mass of Pt in the sample, HER activity of Pt-SA/TiON*_x_* becomes comparable with Pt/C and Pt/TiON*_x_* (not shown), which means that Pt single atoms embedded in TiON*_x_* can serve as active sites for HER. However, it is possible that the TiON*_x_* support also contributes to the measured activity of the Pt-SA/TiON*_x_* sample. In the case that Pt single atoms are active sites for HER, the low activity of Pt-SA/TiON*_x_* can be linked with the low concentration of Pt single atoms in the sample. Translated to Pt/TiON*_x_*, this means that due to the even lower concentration of single atoms, they do not contribute to the overall activity of the catalyst, which rather originates from Pt nanoparticles. Another possible reason for the observed HER enhancement could be a slight difference in particle size distribution for Pt/C and Pt/TiON*_x_* samples. There is no clear consensus about the effect of particle size on the HER activity of Pt-based samples, as quite opposing reports can be found in the literature.^67−69^ To exclude the possible effect of particle size, we measured the HER activities of two more benchmark Pt/C catalysts with an average particle size of 2–3 nm (TEC10E50E from Tanaka) and 5 nm (TEC10E50E-HT from Tanaka). Detailed characterization of these two commercial catalysts can be found in our previous works.^70^ A comparison of HER activities of Pt/C benchmarks, Figure S5, revealed that the activity did not improve with increasing particle size. We, therefore, believe that particle size does not play a significant role in this study. Other possible nonintrinsic factors can also be excluded since the mass of the loaded Pt on the electrode is slightly higher for Pt/C than for Pt/TiON*_x_* (5 *versus* 4.6 μg), and Pt ESA is also in favor of Pt/C (87 *versus* 61 m^2^ g_Pt_^–1^).

**Figure 5:**
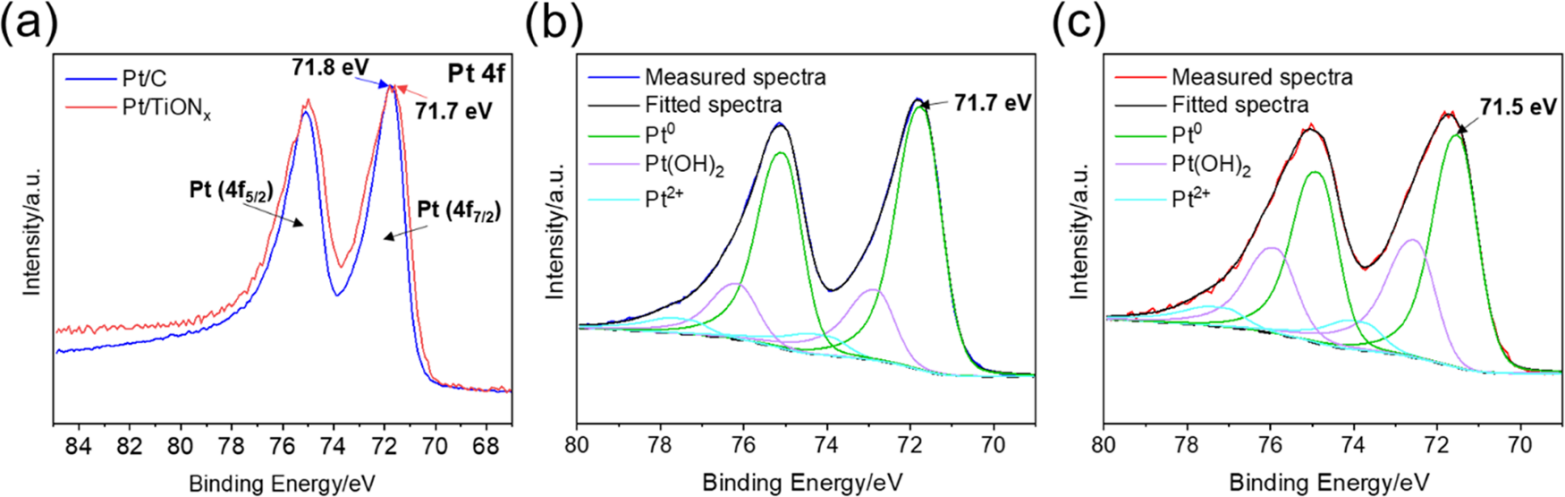
XPS characterization of the Pt 4f region: (a) normalized Pt 4f region for Pt/C and Pt/TiON*_x_*; (b) fitted Pt 4f region for Pt/C; and (c) fitted Pt 4f region for Pt/TiON*_x_*.

Based on the above discussion, we propose that the TiON*_x_* substrate affects the HER performance of supported Pt via SMSI. A similar effect of TiON*_x_* was already reported by our group for supported Ir nanoparticles, which led to the improved activity and stability of Ir/TiON*_x_* composite for OER.^30^ As mentioned earlier, there are a few non-HER-related indications of SMSI: (i) TiON*_x_* is able to anchor Pt single atoms; (ii) CO electro-oxidation proceeds at lower potentials on Pt/TiON*_x_* with respect to Pt/C. To further check the existence of SMSI induced between the TiON*_x_* support and Pt nanoparticles, the XPS spectra of the Pt 4f core level in both Pt/C and Pt/TiON*_x_* were investigated (Figure 5). Characteristic Pt 4f spectra for both Pt/C and Pt/TiON*_x_* samples were fitted with one f doublet with 4f_7/2_ and 4f_5/2_ components (Figure 5a). When the normalized Pt 4f spectra are compared, a slight difference in the position of the Pt 4f_7/2_ peak can be observed. In particular, the Pt 4f_7/2_ peak position for Pt/TiON*_x_* (71.7 eV) is shifted toward lower binding energies (BE) compared to the Pt/C (71.8 eV). This suggests a difference in the electronic interaction of Pt NPs with the C or TiON*_x_* supports, which could be attributed to SMSI in the latter case. However, the shape of the Pt 4f regions indicates the presence of more than just a Pt^0^ species in the samples. By fitting the spectra using the parameters as described in the Section 2, a total of three Pt species (Pt^0^, Pt(OH)_2_, and PtO) were identified in both Pt/C (Figure 5b) and Pt/TiON*_x_* (Figure 5c). The positions of the peaks for Pt^0^, Pt(OH)_2_, and PtO determined for Pt/C are 71.7, 72,8, and 74.0 eV, respectively. The peaks for Pt^0^, Pt(OH)_2_, and PtO for Pt/TiON*_x_* were fitted at 71.5, 72.5, and 73.7 eV, respectively. When the positions of the Pt^0^ peaks for Pt/C and Pt/TiON*_x_* are compared, an even larger shift of 0.2 eV toward lower BE is observed in the Pt/TiON*_x_* sample. The position of the peaks for the other two Pt species follows the same trend. More specifically, this shift of BE indicates a rearrangement in the electron density on Pt caused by the electronic interaction with underlying TiON*_x_*, which can be favorable for the electrocatalytic activity of supported Pt.^71−73^ Overall, the XPS results substantiate our hypothesis that the TiON*_x_* support triggers SMSI with Pt nanoparticles. We thus propose that the improved HER performance of Pt/TiON*_x_* can be attributed to SMSI, which adjusts the hydrogen adsorption energy on Pt active sites (i.e., activity enhancement). Furthermore, the stronger binding of Pt nanoparticles to underlying TiON*_x_* prevents particle detachment and coalescence during prolonged HER cycling (i.e., stability enhancement). These aspects of the SMSI will be further studied by DFT calculations below.

### 3.3 DFT Calculations

**Figure 6:**
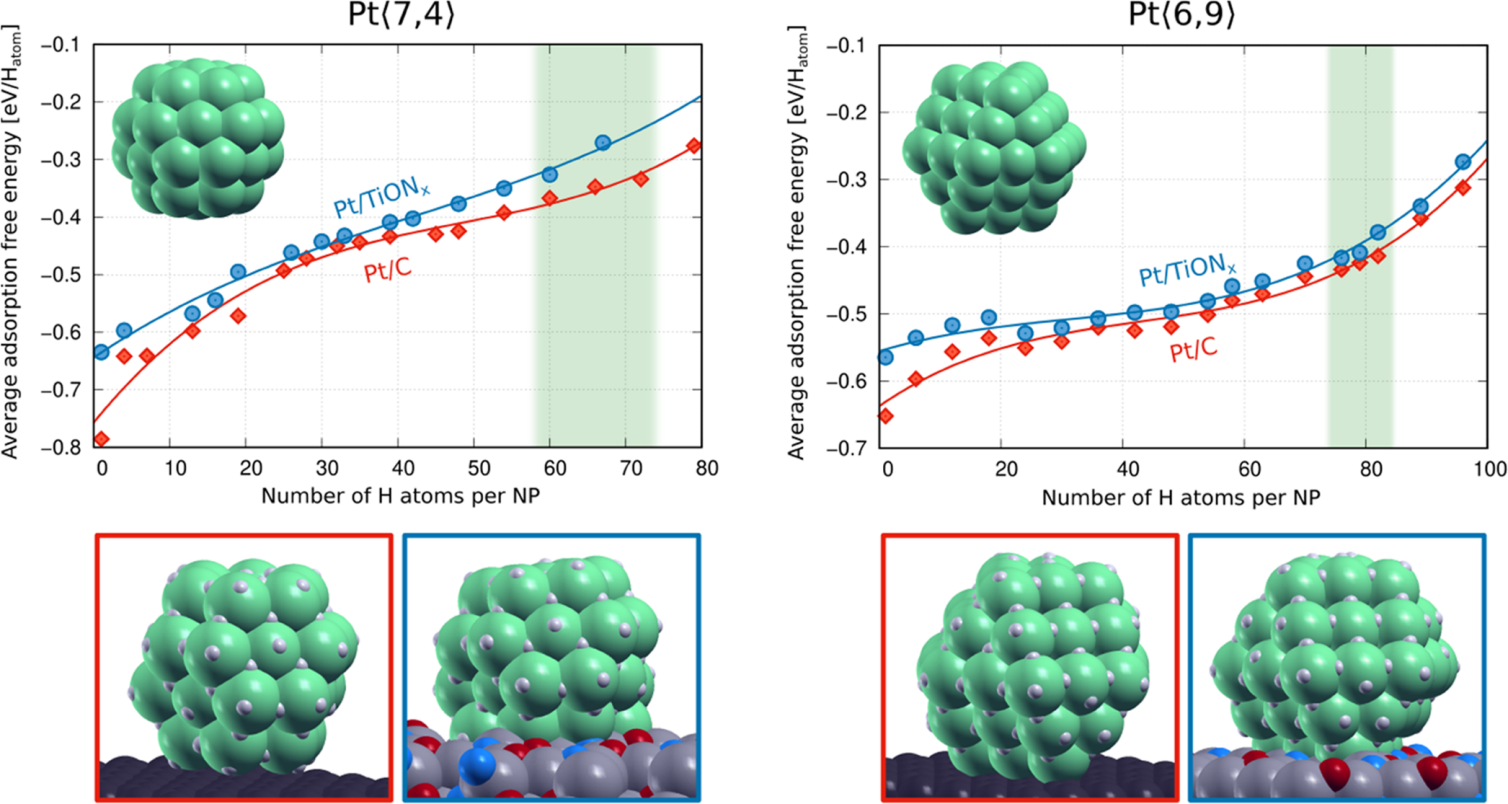
Average hydrogen adsorption free energy (at 298 K and 1 atm) as a function of the number of H atoms on the two Pt nanoparticles supported on TiON*_x_* and graphene. Vertical green stripes indicate H coverages relevant under HER conditions. Only the adsorption free energy of the most stable identified structure for each number of H atoms is reported. Below the graphs, exemplar snapshots of each considered Pt NP/support system at high H coverage are shown (snapshots at other H coverages are provided in Figures S6 and S7 in the Supporting information).

A way to reduce the sensitivity of computational results to a particular nanoparticle is to use several different nanoparticles. As a compromise between reliability, computational cost, and human effort, we performed calculations with two Pt NPs: Pt⟨7,4⟩ and Pt⟨6,9⟩ with a diameter of 1.1 and 1.3 nm, respectively. They were built to match the experimentally observed shapes (Figure S2). A different number of H atoms was adsorbed on the Pt/C and Pt/TiON*_x_* systems, ranging from a single adsorbed H atom to coverages surpassing two H atoms per surface Pt atom. Figure 6 shows the average hydrogen adsorption free energy (at 298 K and 1 atm), calculated with eq 6, as a function of the number of adsorbed H atoms per NP for the two considered Pt NPs (at each number of H atoms, only the most stable identified structure is considered). In general, hydrogen adsorption free energy becomes less exergonic as the coverage increases. However, at a particular number of H atoms, the Pt/C systems consistently exhibit more exergonic adsorption free energy over the whole coverage range: this trend is observed on both Pt NPs. This observation indicates that H atoms bind slightly stronger to Pt/C than Pt/TiON*_x_* when the same number of H atoms per NP is considered in both cases.

To determine which coverages are relevant under experimental conditions, we performed a thermodynamic analysis in terms of adsorption surface free energy as a function of hydrogen chemical potential; HER occurs at electrode potentials between −0.1 and 0 V *versus* RHE (Figure 3a) which, according to eq 11, corresponds to the Δμ_H_ range of 0–0.1 eV. The corresponding results are summarized in Figure S8 in the Supporting information. This analysis reveals that under the relevant range of Δμ_H_, the most stable structures display H coverages around two H atoms per surface Pt atom of isolated NP; vertical green stripes indicate these H coverages in Figure 6. We can thus deduce that the Pt/C system adsorbs H atoms slightly stronger than Pt/TiON*_x_* under conditions relevant to HER. In the HER volcano plot, platinum lies near the top of the volcano, although it slightly overbinds H atoms.^9^ According to our results, the Pt/C system binds H somewhat stronger than Pt/TiON*_x_*, which, according to the volcano plot argument, suggests that Pt/TiON*_x_* should be slightly superior to Pt/C for HER.

**Figure 7:**
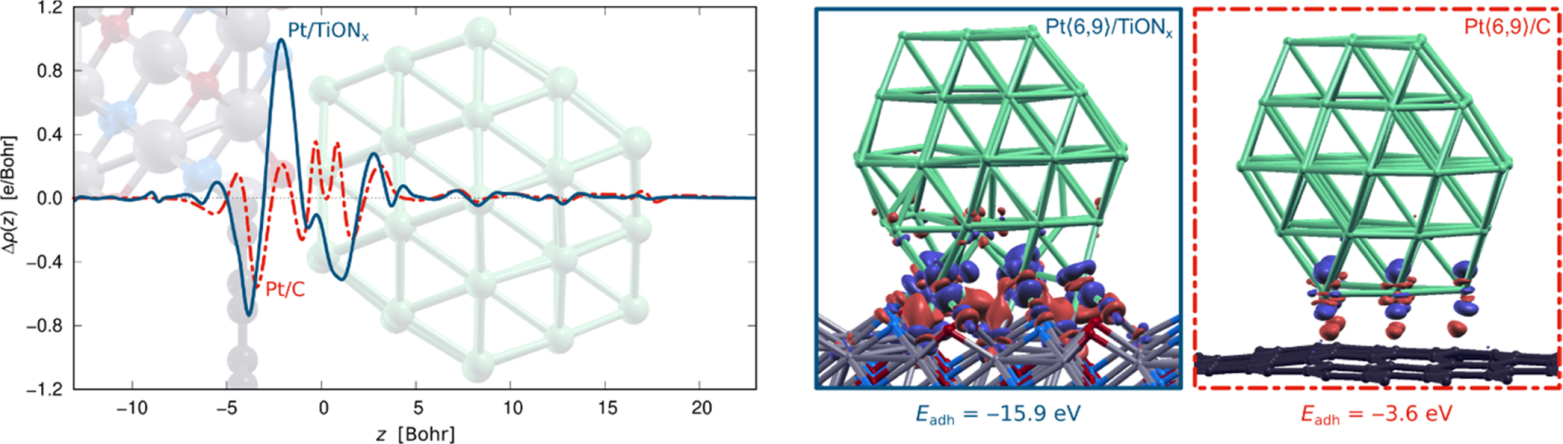
Adhesion of Pt⟨6,9⟩ on TiON*_x_* and graphene supports. Left: planar integrated electron charge density difference, Δρ(*z*) of eq 14, for Pt/TiON*_x_* (blue curve) and Pt/C (red curve); Δ*ρ*(*z*) > 0 corresponds to electron excess. The respective structures are superposed with the Δρ(*z*) curves to facilitate interpretation (TiON*_x_* support is shown in the upper left half, graphene support in the bottom left half); *z* = 0 is arbitrarily set to the bottom layer of Pt⟨6,9⟩. Right: the corresponding 3D electron charge density difference, Δρ(***r***) of eq 13. The Δρ(***r***) plots are drawn with isosurfaces of ±0.01 e Bohr; blue (red) color represents the electron-deficit (excess) regions.

To shed some light on why Pt/TiON*_x_* binds H atoms slightly weaker than Pt/C, we analyzed the adhesion of Pt NPs on the two supports. DFT calculations reveal that the adhesion of Pt NPs is considerably stronger to TiON*_x_* than to graphene. For Pt⟨6,9⟩ the *E*_adh_ values are −15.9 and −3.6 eV on the TiON*_x_* and graphene supports, respectively, and for Pt⟨7,4⟩, the respective values are −14.7 and −2.4 eV. Moreover, electron charge density differences and planar integrated electron charge density differences, shown in Figure 7, reveal a much larger electron charge redistribution at the Pt⟨6,9⟩/TiON*_x_* interface than at the Pt⟨6,9⟩/C interface. Figure 7 thus confirms that TiON*_x_* binds Pt NPs much stronger than graphene. A stronger adhesion provides two benefits for HER: first, it reduces the detachment and coalescence of Pt NPs, thus keeping the Pt NPs well dispersed and the platinum surface area maximized during the reaction. Second, it is known that supports that bind metal NPs stronger affect their reactivity such that NPs bind small adsorbates weaker, provided that adsorbates bind mostly covalently.^74^

## 4 Conclusions

In the present contribution, we studied the HER activity of a novel composite catalyst consisting of Pt nanoparticles supported on TiON*_x_*, which in turn had been dispersed over reduced graphene oxide nanoribbons to maximize its surface area. Characterization of Pt/TiON*_x_* revealed the presence of small-sized (2–3 nm in diameter) Pt nanoparticles, which were well distributed over the TiON*_x_* support. Pt/TiON*_x_* outperformed the benchmark Pt/C catalyst in terms of both HER activity and stability in an acid electrolyte, which we ascribed to the SMSI effect of the underlying TiON*_x_*. XPS revealed that TiON*_x_* support affects the electronic states of Pt nanoparticles, which can be correlated with SMSI. DFT calculations confirmed SMSI and revealed that the TiON*_x_* substrate tunes adsorption energetics of intermediate hydrogen species and anchors Pt nanoparticles significantly stronger than carbon, thus improving the HER activity and stability of supported Pt. This work suggests that using TiON*_x_* instead of carbon-based substrates, the intrinsic activity and stability of Pt (and possibly other electrocatalysts) for HER (and other reactions) can be improved via the beneficial effect of the TiON*_x_* support on the catalyst’s electronic structure.

## Supporting Information Available

Additional TEM characterization of Pt/C, Pt/TiON*_x_*, and Pt-SA/TiON*_x_* samples; HER measurements on different Pt/C benchmarks and Pt-SA/TiON*_x_*; snapshots at different H coverages used for DFT; and thermodynamic analysis of the adsorption surface free energy as a function of the hydrogen chemical potential (PDF)
