# Supplementary material for: Improving the HER Activity and Stability of Pt Nanoparticles by Titanium Oxynitride Support
Source: ACS Catal. 2022 Oct 12;12(20):13021–33. doi: 10.1021/acscatal.2c03214 (PMC9594320; doi:10.1021/acscatal.2c03214)
Supplement: Supplementary file 1 — cs2c03214_si_001.pdf [file cs2c03214_si_001.pdf]

Supporting Information

Improving the HER Activity and Stability of Pt Nanoparticles by Titanium Oxynitride Support

Milutin Smiljanić[a,b*], Stefan Panić[a], Marjan Bele[a], Francisco Ruiz-Zepeda[a], Luka Pavko[a], Lea Gašparič[c,d,e], Anton Kokalj[c,d], Miran Gaberšček[a,f], Nejc Hodnik[a,d,g*]

[a]*Department of Materials Chemistry, National Institute of Chemistry*

Hajdrihova 19, 1000 Ljubljana, Slovenia

[b]*Laboratory for Atomic Physics, Institute for Nuclear Sciences Vinča, University of Belgrade, Mike Alasa 12-14, 11001 Belgrade, Serbia*

[c]*Department of Physical and Organic Chemistry, Jožef Stefan Institute, Jamova cesta 39, 1000 Ljubljana, Slovenia*

[d]*Jožef Stefan International Postgraduate School, Jamova cesta 39, 1000 Ljubljana, Slovenia*

[e]*Centre of Excellence for Low-Carbon Technologies, Hajdrihova 19, Ljubljana, Slovenia*

[f]*University of Ljubljana, Faculty of Chemistry and Chemical Technology, Večna pot 113, 1000 Ljubljana, Slovenia*

[g]*University of Nova Gorica, Vipavska 13, 5000 Nova Gorica, Slovenia*

[*]Corresponding authors: milutin.smiljanic@ki.si; nejc.hodnik@ki.si

Characterization of benchmark Pt/C catalyst

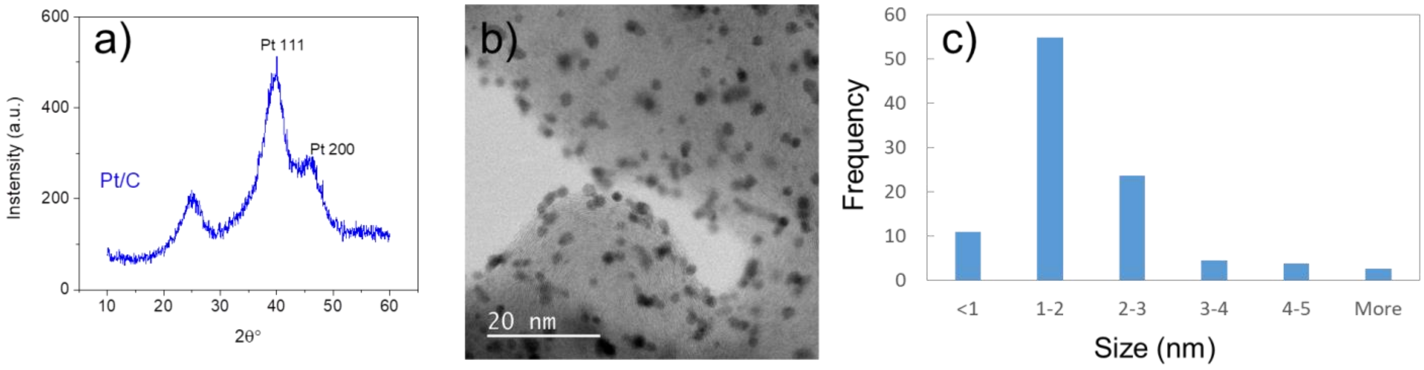

Figure S1. Characterization of a Pt/C sample (Premetek, 20 wt.%): (a) XRD spectra; (b) TEM imaging; and (c) corresponding particle size distribution.

STEM analysis of Pt nanoparticles from Pt/C sample

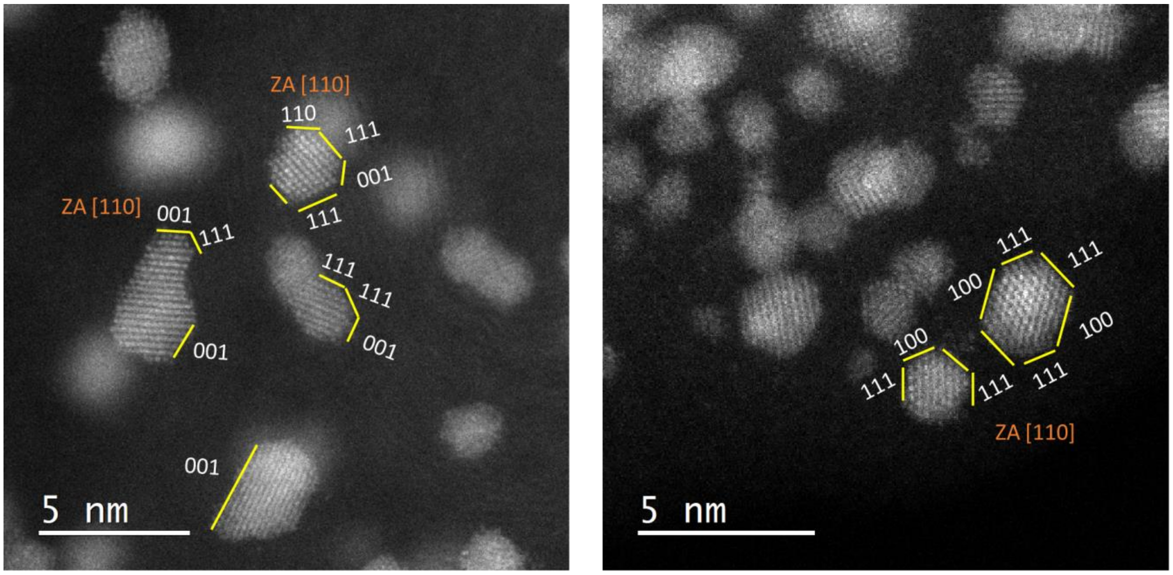

Figure S2. STEM analysis of Pt nanoparticles present in Pt/C. The same low miller index facets were observed on Pt nanoparticles of the Pt/$TiON_x$ sample.

Presence of Pt single atoms in Pt/TiONx composite

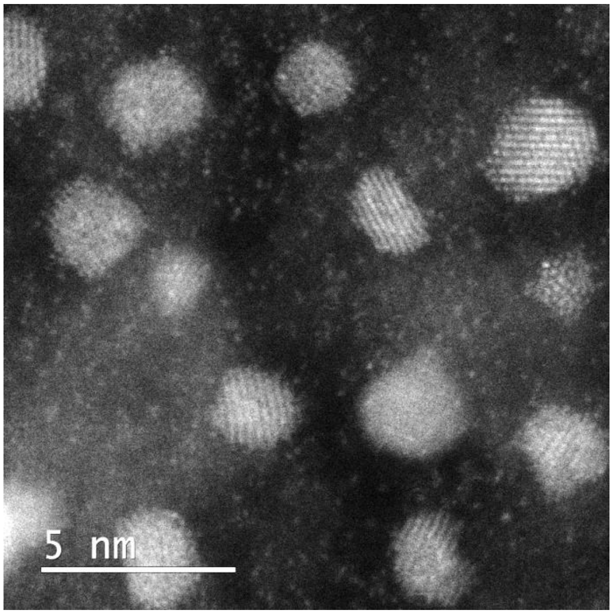

Figure S3. High magnification STEM image of the Pt/$TiON_x$ composite showing the presence of Pt single atoms anchored on $TiON_x$. Single atoms are represented by bright dots surrounding Pt nanoparticles.

Characterization of Pt single atoms supported on $TiON_x$ and investigations of HER catalytic activity

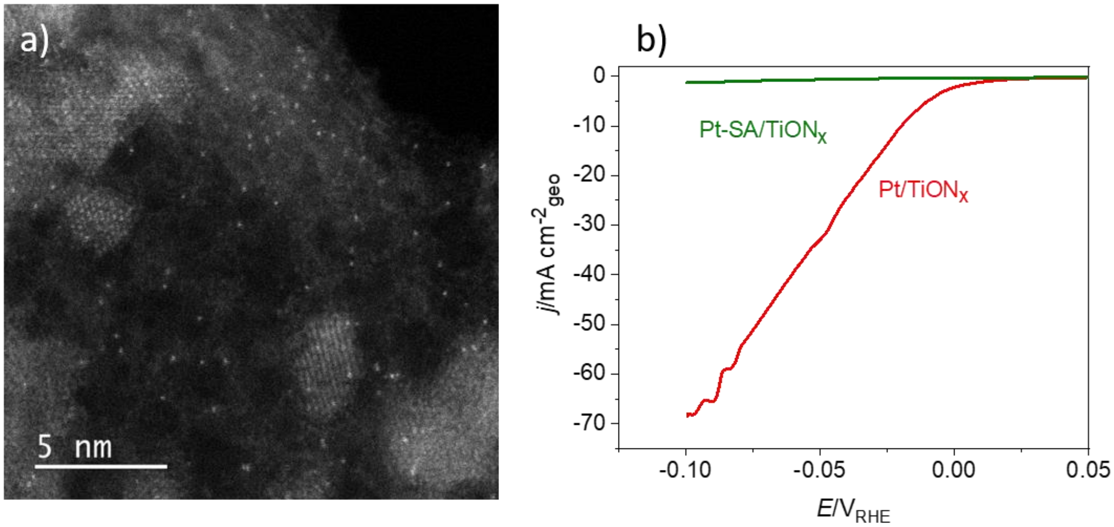

Figure S4. (a) STEM imaging of the Pt-SA/$TiON_x$ sample confirming the presence of only Pt single atoms anchored on $TiON_x$; (b) Comparison of the HER activities of the Pt-SA/$TiON_x$ and Pt/$TiON_x$ samples (10 mV/s, 0.1 M $HClO_4$)

Comparison of the HER reactivity of Pt/C benchmarks with different average particle size

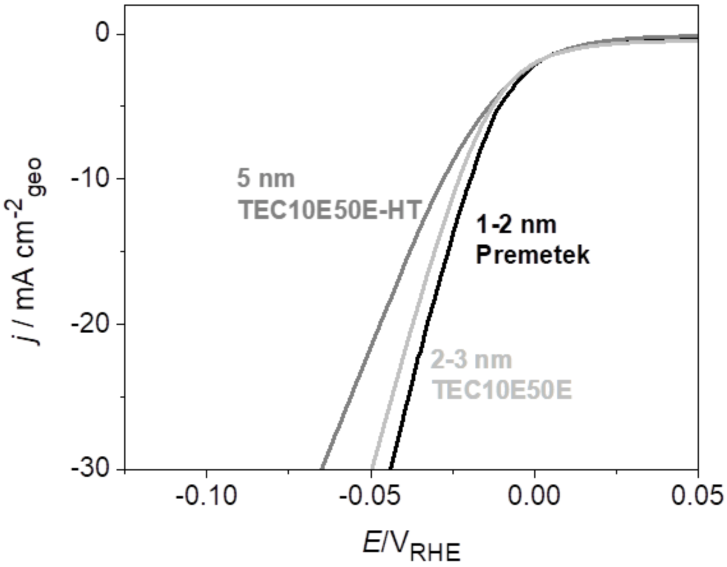

Figure S5. Comparison of the HER activities of different Pt/C benchmark catalysts. Polarization curves were recorded in Ar-saturated 0.1 M $HClO_4$ electrolyte at a scan rate of 10 mV/s, while Pt loading was set to 5 µg.

DFT calculations

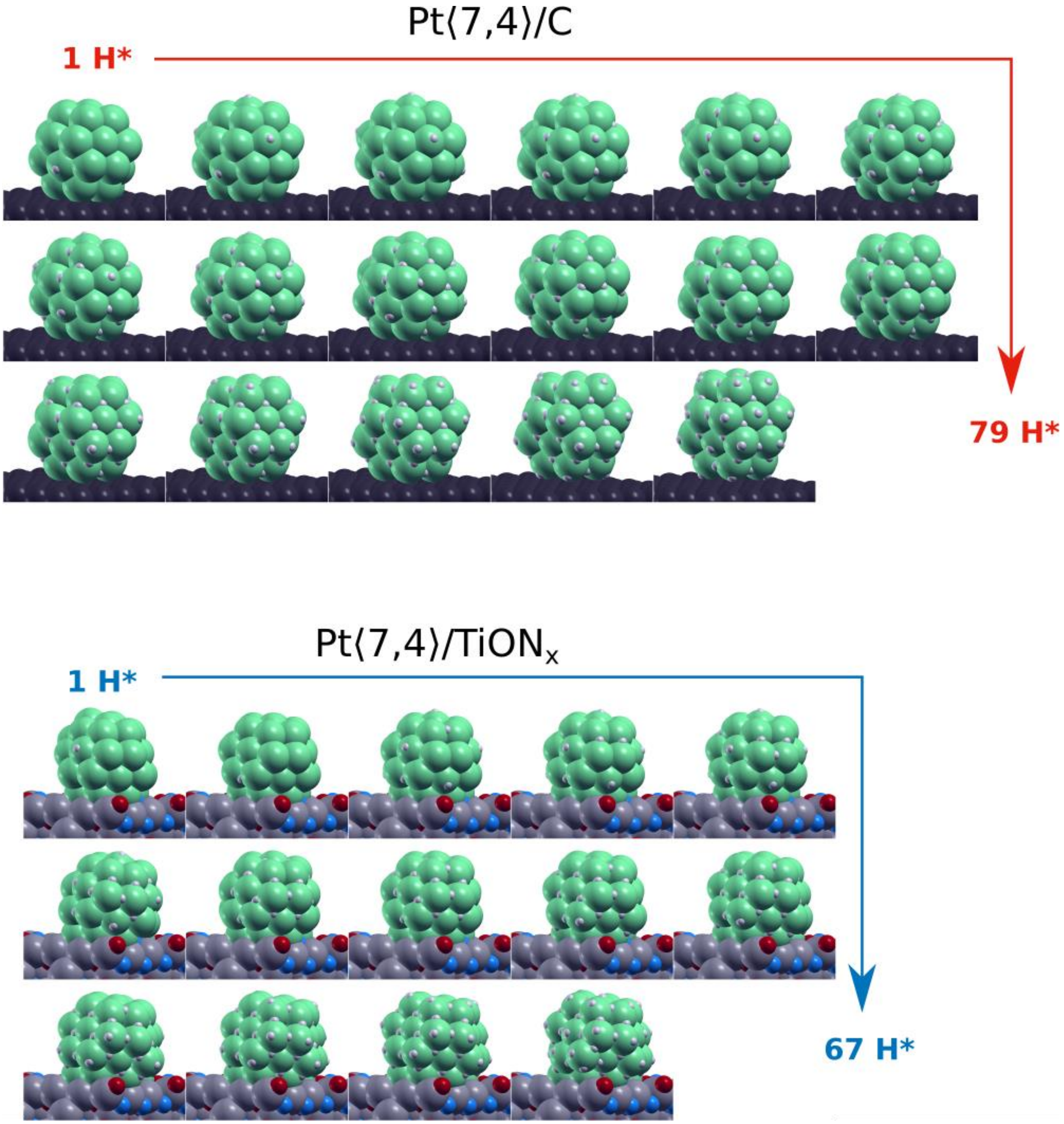

Figure S6. Snapshots of the most stable identified Pt⟨7,4⟩/C and Pt⟨7,4⟩/$TiON_x$ structures as the coverage increases from 1 H atom per Pt⟨7,4⟩ to fully covered Pt⟨7,4⟩ nanoparticle.

DFT calculations

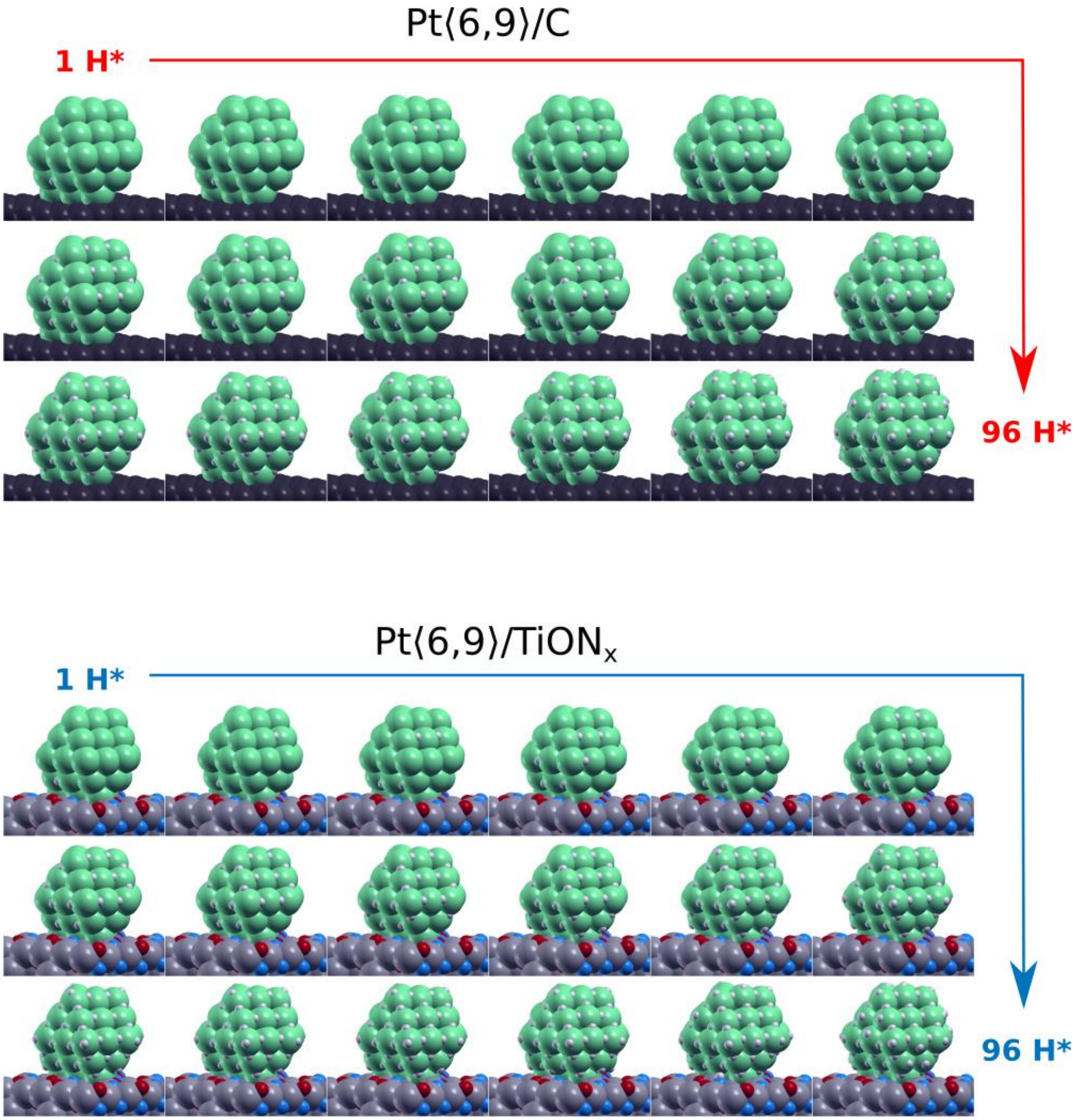

Figure S7. Similar to Figure S6, but for the Pt⟨6,9⟩ nanoparticle.

DFT calculations

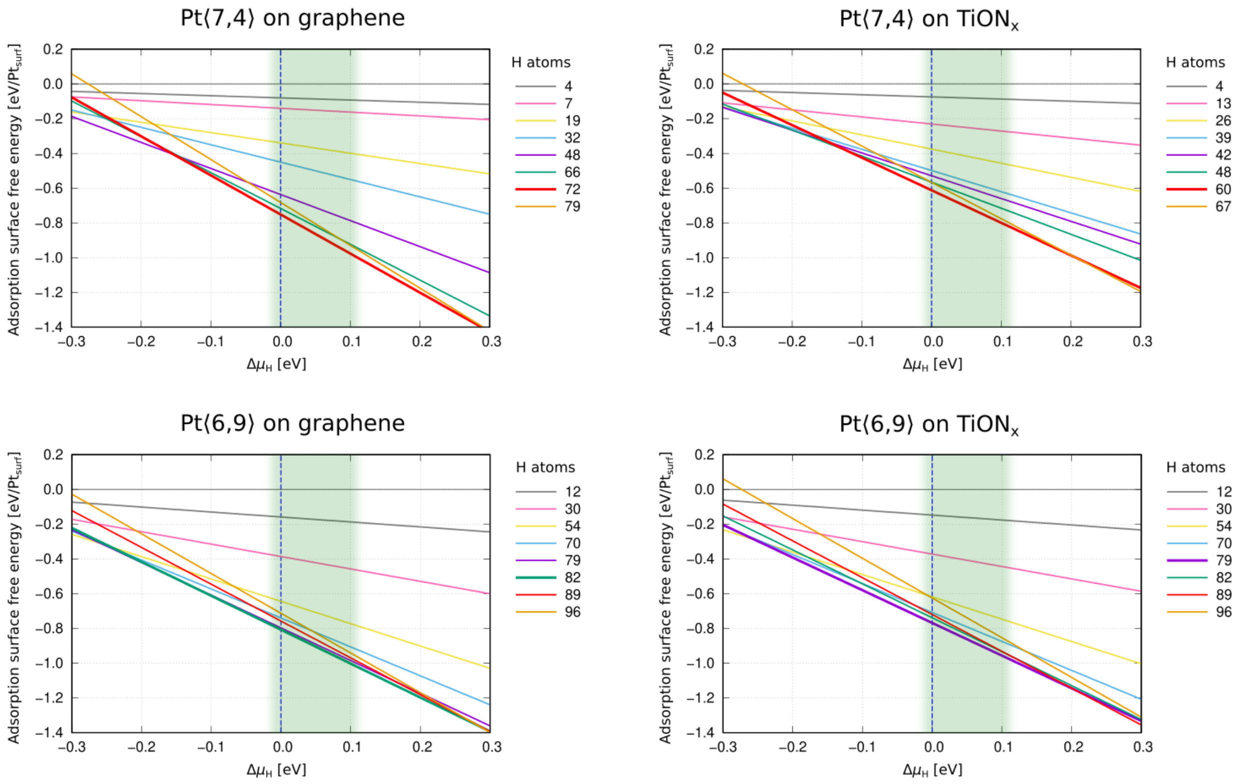

Figure S8. Adsorption surface free energy, γ_{ads} of eq (9) in the main article, versus hydrogen chemical potential, $\Delta\mu_H$ of eq (10). Green background stripes represent the experimentally relevant HER conditions. HER occurs at electrode potentials between –0.1 and 0 V versus RHE (Figure 3), which, according to eq (11) in the main article, corresponds to the $\Delta\mu_H$ range from 0 to 0.1 eV. For Pt⟨7,4⟩, the most stable structures at HER conditions contain 72 and 60 H atoms per NP for Pt/C and Pt/TiON$_x$, respectively, whereas for Pt⟨6,9⟩, the most stable structures contain 82 and 79 H atoms per NP for Pt/C and Pt/TiON$_x$, respectively. Note, however, that other high-coverages around the most stable one are of similar stability under HER conditions.
